# Clinical Features and Prognoses of IgG4-Positive and IgG4-Negative Lacrimal Lymphomas

**DOI:** 10.3389/fonc.2021.622847

**Published:** 2021-03-08

**Authors:** Rui Liu, Jinjin Wang, Nan Wang, Jing Li, Xin Ge, Jingxue Zhang, Jianmin Ma

**Affiliations:** Beijing Ophthalmology and Visual Sciences Key Laboratory, Beijing Tongren Eye Center, Beijing Tongren Hospital, Capital Medical University, Beijing, China

**Keywords:** IgG4-positive, lacrimal gland, lymphoma, clinical features, prognosis

## Abstract

**Purpose:** The clinical characteristics and prognoses of immunoglobulin G4-positive (IgG4+) and IgG4-negative (IgG4−) lacrimal lymphomas were comparatively analyzed to screen for clinical indicators with differential diagnostic significance.

**Methods:** This was a retrospective and single-center clinical study. From June 2011 to June 2018, clinical data of 39 patients with lacrimal lymphoma, diagnosed by histopathology were collected from the Department of Ophthalmology, Beijing Tongren Hospital, Capital Medical University.

**Results:** According to inclusion and exclusion criteria, 30 (76.9%) were in the IgG4− group and 9 (23.1%) were in the IgG4+ group. In the IgG4+ group, the sex ratio of male to female was 2:1 and the mean age was 56.67 ± 13.29 years old. In the IgG4− group, the sex ratio of male to female was 3.29:1 and the mean age was 61.47 ± 12.87 years old. Statistical analysis of the clinical indicators showed significant differences between the two groups in affected eye, preoperative history of glucocorticoids, ocular nerve thickening, the expression of serum IgG4 and prognosis (*P* < 0.05). There was no significant statistical difference in laboratory indicators between the two groups, including C3, C4, RF, ASO, CRP, IgA, IgM, IgG, IgG1, IgG2, and IgG3 (*P* > 0.05). The event-free cumulative percentages at 3 and 5 years for the 39 lacrimal lymphomas were 81.1 and 62.4%, respectively, with recurrence and death as end events. In 34 lacrimal gland MALT lymphoma cases, the event-free cumulative percentages at 3 and 5 years were 84.4 and 69.1%, respectively. In the IgG4+ and IgG4− groups, the event-free cumulative percentages at 3 years were 75.0 and 87.7%, respectively. The event-free cumulative percentage at 5 years was 62.6% in the IgG4-group and insignificant in the IgG4+ group. There was no statistical difference in event-free cumulative percentage between the two groups (*P* = 0.983).

**Conclusion:** The pathogenesis and disease characteristics of IgG4-positive lacrimal lymphoma may differ from IgG4-negative lacrimal lymphoma, but the positive expression of IgG4 may not have significant influence on the recurrence of lacrimal lymphoma.

## Introduction

Ocular adnexal lymphoma can involve multiple tissues, such as the conjunctiva and lacrimal gland, and accounts for 5–15% of extranodal lymphomas (1, 2). Among these, mucosa-associated lymphoid tissue (MALT) lymphoma is the most common pathological type, accounting for about 50–70% of ocular adnexal lymphoma, and has been found to be as high as 90% (3, 4). IgG4-related ocular disease can involve all orbital tissues and is characterized by extensive lymphocyte infiltration and sclerosis formation (5). Studies have found that the presence of a large number of IgG4+ plasma cells can be detected in lymphoma, suggesting a certain relationship between lymphoma and IgG4-related ocular disease (6, 7). At present, the role and mechanism of IgG4-positive expression in the pathogenesis of lymphoma are still unclear.

In this study, the pathological features, laboratory indicators, treatment, and prognosis of patients with IgG4+ and IgG4− lymphomas were compared and analyzed in order to find clinical and laboratory indicators with differential diagnosis significance.

## Methods

### Patients

The current study is a retrospective, single-center clinical study conducted between June 2011 and June 2018. The medical records of lacrimal lymphoma confirmed by pathology were collected from the medical records database of the Department of Ophthalmology, Beijing Tongren Hospital, Capital Medical University, by professional ophthalmologists. The number of IgG4 positive expression was counted and confirmed by two professional pathologists. Since some patients had a history of IgG4-related ocular disease before the diagnosis of lymphoma and were treated with glucocorticoids, the inclusion criteria for IgG4+ lymphoma in this study were as follows: (1) diagnosed as lymphoma by histopathology; (2) histopathological findings > 10 IgG4+ plasma cells per high power field (HPF) and an IgG4+/IgG+ cell ratio > 30%; (3) a serum IgG4 concentration > 135 mg/dl; and (4) the patient had a history of IgG4-related ocular disease clearly diagnosed by pathology. Inclusion criteria for IgG4− lymphoma were as follows: (1) diagnosed as lymphoma by histopathology; (2) histopathological findings ≤ 10 IgG4+ plasma cells per HPF and an IgG4+/IgG+ cell ratio ≤ 30%; (3) a serum IgG4 concentration ≤ 135 mg/dL; (4) no history of IgG4-related ocular disease. Exclusion criteria: (1) other ocular lymphoproliferative lesions; (2) Other lacrimal tumors, including lacrimal pleomorphic adenoma, adenoid cystic carcinoma, and adenocarcinoma. A total of nine IgG4+ lymphomas (IgG4+ group) and 30 IgG4− lymphomas (IgG4− group) were identified that met two or more inclusion criteria. The study was approved by the Institutional Review Board of Beijing Tongren Hospital and carried out according to the tenets of the Declaration of Helsinki.

### Clinical Data Collection

General information was collected from medical records database, including medical record number, age, gender, affected eye, previous history (including preoperative glucocorticoid therapy, operations, and sinusitis), clinical manifestations, course, imaging findings, immunohistochemical indicators, and prognosis. Imaging findings, including magnetic resonance imaging (MRI) or computed tomography (CT), were obtained and confirmed by professional radiologists. Course refers to the duration of clinical symptoms. The cases that did not meet the inclusion criteria and had incomplete medical history were excluded by two professional ophthalmologists.

### Laboratory Data Collection

Peripheral venous blood samples were collected from the two groups of patients. The enzyme-linked immunosorbent assay was used to test indicators, including complement C3 and C4, rheumatoid factor (RF), c-reactive protein (CRP), hemolysin against streptococcus O (ASO), IgA, IgM, IgG, and IgG-subtypes (IgG1, IgG2, IgG3, IgG4). Enzyme-linked immuno sorbent assay (ELISA) kits were purchased from eBioscience (USA). The 96-well microplates were coated with captured antibodies. Plasma samples and standards were added and incubated for 2 h. The wells were washed and HRP-conjugated detection antibodies were added into each well. Plates were washed three times, and the reactions were stopped after incubation with the TMB substrate reagent and optical density read at 450 nm using a micro-plate reader (PerkinElmer, USA).

### Treatment and Prognosis

All patients were treated with surgical resection. Lesions were removed via the anterior temporal eyebrow arch skin incision or the double eyelid skin incision depending on the size of the lesion. Adjuvant therapy, including radiotherapy and chemotherapy, were selected depending on the disease. The appropriate radiation range or chemotherapy regimen was chosen according to the pathological type of tumor and the extent of lesion involvement. The common methods used were external gamma radiation therapy and the CHOP regimen, which included cyclophosphamide, doxorubicin, vincristine, and prednisone.

The effective follow-up time was from definitive diagnosis of the first biopsy to the death of the patient or June 2020. The observation indicators during follow-up were general condition, including eyesight, eyelid swelling, and proptosis and imaging findings. In order to objectively evaluate the therapeutic effect and observe whether the lesions had recurred, preoperative and postoperative MRI examination was required for each patient. The specific time points were pre-surgery, 6 months, 1–3 years, and 5 years post-surgery. CT scans were done in patients with contraindications to MRI. Criteria for recurrence was as follows: eyelid swelling or lacrimal gland enlargement, accompanied by objective imaging evidence of lacrimal gland involvement, and confirmed by histopathological examination.

### Statistical Analysis

GraphPad Prism 8.0 and SPSS 25.0 software were used for analysis. Measurement data were tested by One-sample Kolmogorov-Smirnov test. Mean ± standard deviation and independent sample *t* test were used to test the data of two groups consistent with normal distribution. Median and non-parametric rank sum test were used for data that did not conform to normal distribution. The chi-square test or Fisher's exact test were used for counting data. Survival curves for events were generated using the Kaplan–Meier method and compared between the two groups using log-rank tests. The influencing factors were analyzed via binary Logistic regression analysis. A *P*-value < 0.05 was considered statistically significant.

## Results

### Results of Clinical Characteristics Analysis

The gender, age, affected eye, and previous histories of the 39 lymphoma patients were retrospectively analyzed. The results (Table 1) showed that the male-female ratio was 2:1 in the IgG4+ group and 3.29:1 in the IgG4− group. The mean age was 56.67 ± 13.29 years in the IgG4+ group and 61.47 ± 12.87 years in the IgG4− group. There was no statistically significant difference in the male to female ratio (*P* = 0.669) or age (*P* = 0.336) between the two groups. There was one case of right eye, five cases of left eye, and three cases of binocular disease in the IgG4+ group. There were 15 cases of right eye, 11 cases of left eye, and four cases of binocular disease in the IgG4− group. The incidence of monocular disease was more common in both groups and IgG4− group had a higher significantly incidence of monocular disease (*P* = 0.031). IgG4+ group had a significantly higher incidence of preoperative glucocorticoid therapy (*P* = 0.003). In the IgG4+ group, three patients had a history of previous surgery and three patients had a history of sinusitis. In the IgG4− group, two patients had a history of sinusitis. There were no statistically significant differences in history of previous surgery or sinusitis between the two groups (*P* > 0.05).

**Table 1:** Clinical characteristics of the IgG4+ and IgG4− lacrimal lymphoma.

**Data**	**IgG4+ (*n* = 9)**	**IgG4− (*n* = 30)**	**Test value**	***P***
**GENDER**				
Male	6	23	–	0.669*
Female	3	7		
**Age (years)**	56.67 ± 13.29	61.47 ± 12.87	−0.974	0.336^&^
**LATERALITY**				
Right	1	15	–	0.031*
Left	5	11		
Bilateral	3	4		
**PREVIOUS HISTORY**				
Glucocorticoid	7	2	–	0.003*
Operation	3	0	–	0.500*
Sinusitis	3	2	–	0.070*
**CLINICAL MANIFESTATIONS**				
Eyelid swelling	7	13	–	0.127*
Proptosis	2	9	–	1.000*
Eyelid mass	1	9	–	0.400*
Decreased vision	0	1	–	1.000*
Diplopia	0	1	–	1.000*
Dry eye	0	1	–	1.000*
Tearing	1	2	–	0.556*
Pain	0	1	–	1.000*
**IMAGING FINDINGS**				
Lacrimal gland enlargement	9	30	NA	NA
Sinus mucosa thickening	3	2	–	0.070*
Nerve thickening	2	0	–	0.049*
Extraocular muscle thickening	3	3	–	0.123*
Bone destruction	0	1	–	1.000*
**Course (months)**	26.00 ± 33.99	20.57 ± 25.10	0.524	0.603^&^
**LABORATORY INDICATORS (MG/DL)**				
C3 (900–1800)	1,025.46 ± 221.73	1,020.31 ± 199.44	0.066	0.948^&^
C4 (100–400)	238.74 ± 62.04	220.09 ± 60.34	0.808	0.424^&^
RF (0–20)	7.20	5.76	−0.417	0.682^#^
ASO (0–200)	39.00	58.55	−1.000	0.332^#^
CRP (0–5)	0.92	1.08	−0.133	0.909^#^
IgA (0.7–4)	1.97 ± 0.84	2.10 ± 0.86	−0.401	0.690^&^
IgM (0.4–2.3)	1.50	1.57	0.067	0.961^#^
IgG (751–1560)	1,387.78 ± 325.41	1,207.53 ± 228.12	1.879	0.068^&^
IgG1 (382–930)	667.33 ± 180.99	628.71 ± 152.94	0.637	0.528^&^
IgG2 (242–700)	574.00	377.00	−1.883	0.059^#^
IgG3 (22–176)	35.00	54.50	−1.033	0.315^#^
IgG4 (4–87)	126.40	33.00	−3.834	**0.000**^**#**^
**TREATMENT**				
Surgery	1	5	1.313	0.596*
Surgery + glucocorticoid	2	10		
Surgery + radiotherapy	4	8		
Surgery + chemotherapy	2	7		
**Follow-up (years)**	4.03 ± 1.75	3.48 ± 1.44	1.252	0.215^&^
**Prognosis**				
Lost to follow-up	0	4	8.703	0.009*
Recurrence	2	3		
No recurrence	7	17		
Natural death	0	6		

“&” stands for t test, and “*” stands for Fisher's exact test, “#” stands for non-parametric rank sum test, P < 0.05 is statistically significant. NA, Not Applicable. Bold denotes P < 0.05.

**Figure 1:**
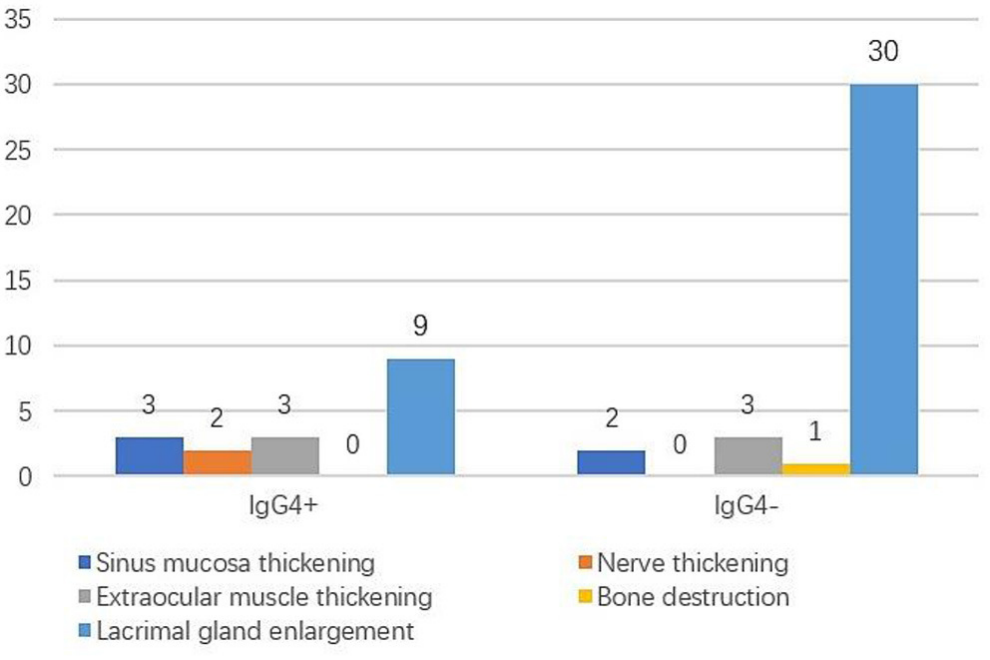
Imaging findings of lymphoma.

The clinical manifestations in the IgG4+ group were eyelid swelling in seven cases (77.8%), proptosis in two cases (22.2%), eyelid mass in one case (11.1%), and tearing in one case (11.1%). The clinical manifestations in the IgG4− group were eyelid swelling in 13 cases (43.3%), proptosis in nine cases (30.0%), eyelid mass in nine cases (30%), tearing in two cases (6.7%), dry eye in one case (3.3%), decreased vision in one case (3.3%), pain in one case (3.3%), and diplopia in one case (3.3%). Eyelid swelling was the main clinical manifestation in both groups and was not statistically significantly different between the two groups (*P* > 0.05). Imaging findings are shown in Figure 1. Lacrimal gland enlargement (100%), extraocular muscle thickening (33.3%), thickening of sinus mucosa (33.3%), and ocular nerve thickening (22.2%) were observed in the IgG4+ group, and lacrimal gland enlargement (100%), extraocular muscle thickening (10%), and sinus mucosa thickening (6.7%) were observed in the IgG4− group. There was a statistically significant difference in ocular nerve thickening between the two groups (*P* = 0.049); however, there was no statistically significant difference in sinus mucosa or extraocular muscle thickening (*P* = 0.070, *P* = 0.123). The mean course was 26.00 ± 33.99 months in the IgG4+ group and 20.57 ± 25.10 months in the IgG4− group and was not statistically different between the two groups (*P* = 0.603).

### Results of Laboratory Indicators Analysis

**Figure 2:**
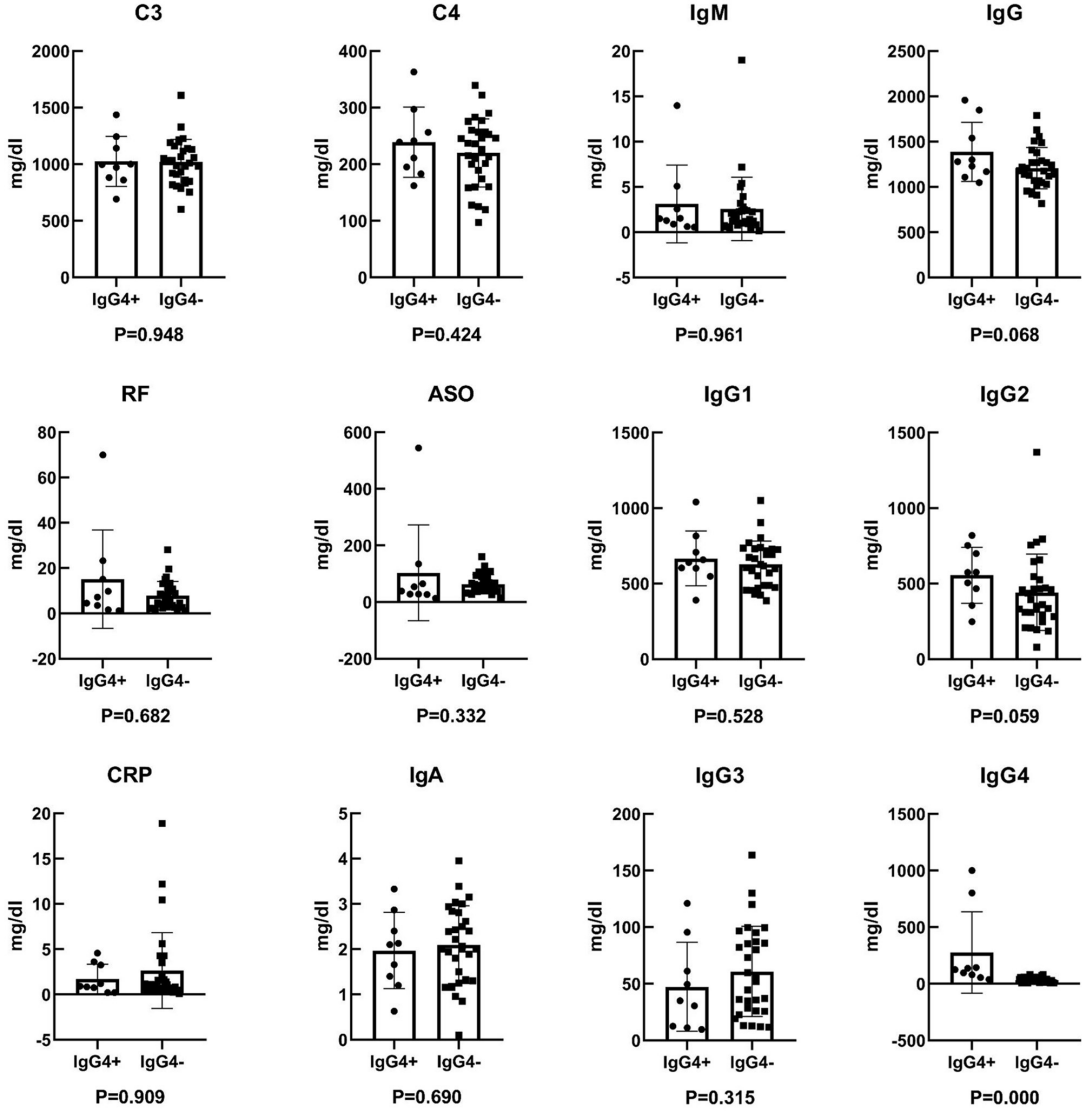
Comparative analysis of laboratory indicators between IgG4+ and IgG4− lymphoma. Comparison of C3 concentration, *P* = 0.948; comparison of C4 concentration, *P* = 0.424; comparison of RF concentration, *P* = 0.682; comparison of ASO concentration, *P* = 0.332; comparison of CRP concentration, *P* = 0.909; comparison of IgA concentration, *P* = 0.690; comparison of IgM concentration, *P* = 0.961; comparison of IgG concentration, *P* = 0.068; comparison of IgG1 concentration, *P* = 0.528; comparison of IgG2 concentration, *P* = 0.059; comparison of IgG3 concentration, *P* = 0.315; comparison of IgG4 concentration, *P* = 0.000. *P* < 0.05 indicates statistical significance.

Twelve laboratory indicators, including C3, C4, RF, ASO, CRP, IgA, IgM, IgG, IgG1, IgG2, IgG3, and IgG4, were analyzed in the 39 patients via the ELISA method. According to statistical analysis, C3, C4, IgA, IgG, and IgG1 were normal distribution, while IgM, RF, ASO, CRP, IgG2, IgG3, and IgG4 were not normal distribution. The results are shown as shown in Figure 2 and Table 1. There was no significant statistical difference in laboratory indicators between the two groups, including C3, C4, RF, ASO, CRP, IgA, IgM, IgG, IgG1, IgG2, and IgG3 (*P* > 0.05). And the expression of serum IgG4 was higher in IgG4+ lymphoma than IgG4− lymphoma (*P* = 0.000).

### Results of Treatment and Outcome Analysis

There were 39 cases of lymphoma, including two NK/T cell lymphomas, two diffuse large B cell lymphomas, one mantle cell lymphoma, and 34 MALT lymphomas. In the IgG4+ group, one case underwent surgical resection, two cases underwent surgery combined with glucocorticoid therapy, four cases underwent surgery combined with radiotherapy, and two cases underwent surgery combined with chemotherapy. In the IgG4− group, five cases underwent surgery, 10 cases underwent surgery and glucocorticoid therapy, eight cases underwent surgery and radiotherapy, and seven cases underwent surgery and chemotherapy. In the IgG4+ group, the mean follow-up time was 4.03 ± 1.75 years and included two cases of recurrence, six cases of no recurrence, and one case of loss to follow-up. The mean follow-up time in the IgG4− group was 3.48 ± 1.44 years and included three cases of recurrence, 17 cases of no recurrence, four cases of loss to follow-up, and six cases of death (two cases of NK/T cell lymphoma, one case of diffuse large B-cell lymphoma, one case of mantle cell lymphoma, and two cases of MALT lymphoma). The results showed that there was a significant difference in prognosis between the IgG4+ and the IgG4− groups (*P* = 0.009), which may have been due to five cases of lymphoma with a high degree of malignancy included in the IgG4− group.

**Figure 3:**
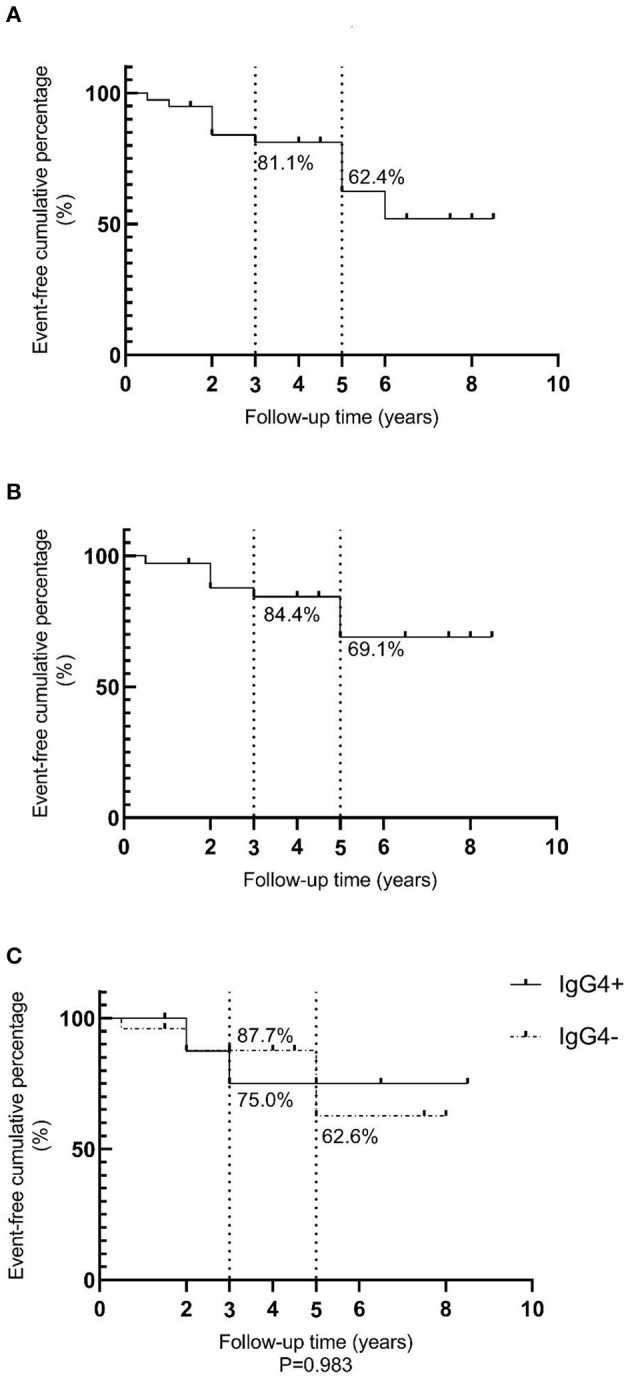
Event-free survival analysis for lymphomas. **(A)** A total event-free survival analysis of 39 lymphomas; **(B)** A total event-free survival analysis of 34 MALT lymphomas; **(C)** Comparative analysis of event-free survival in nine IgG4+MALT lymphomas and 25 IgG4-MALT lymphomas, *P* = 0.983.

An event-free survival analysis was performed, with recurrence and death as endpoint events, as shown in Figure 3A. The event-free cumulative percentages for 3 and 5 years were 81.1 and 62.4%, respectively. Since NK/T cell lymphoma, diffuse large B cell lymphoma, and mantle cell lymphoma with higher degrees of malignancy influenced the prognosis, non-MALT lymphoma cases were excluded. A total of 34 MALT lymphomas were then analyzed and the results are shown in Figures 3B,C. The event-free cumulative percentages for 3 and 5 years were 84.4 and 69.1%, respectively. The event-free cumulative percentages for 3 years in the IgG4+ and IgG4− MALT lymphoma groups were 75.0 and 87.7%, respectively. The event-free cumulative percentage for 5 years in the IgG4− group was 62.6%, while the event-free cumulative percentage for 5 years in the IgG4+ group was insignificant, which may be related to the small sample size and insufficient follow-up time. There was no statistically significant difference between the IgG4+ and IgG4− groups (*P* = 0.983).

## Discussion

In recent years, the relationship between lymphoma and IgG4-related disease (IgG4-RD) has garnered extensive attention of researchers at home and abroad. Some patients with lymphoma, especially MALT lymphoma, have previous histories of IgG4-RD or significantly increased expression levels of IgG4, which are found in the serum and when doing histopathology in primary lymphoma. Thus, it is speculated that IgG4-RD can develop into lymphoma and IgG4 may be involved in the pathogenesis of lymphoma. Oleś (7) et al. considered that lymphoma could occur in the background of IgG4-RD, and that IgG4 may be produced by lymphoma cells. Sohn (8) et al. studied 30 cases of ocular adrenal lymphoma and found that 43.3% were IgG4+ plasma cell MALT lymphomas and 69% had conjunctival involvement. Furthermore, IgG4+ MALT lymphoma had a significantly higher incidence of bilateral ocular addendum involvement and a higher recurrence rate than IgG4− lymphoma (8). Go (9) et al. reported two cases of IgG4-RD that developed into MALT lymphoma. Cheuk (10) et al. reported that three cases of IgG4-associated chronic sclerosing dacryoadenitis developed into lymphoma. In addition, it has been reported that IgG4-RD can develop into diffuse large B-cell lymphoma and follicular lymphoma (10–12). Therefore, IgG4-RD has been found to develop into different pathological types of lymphoma, with MALT lymphoma the most common, though the incidence was low. Since IgG4-RD is a systemic disease, we should notice that lymphoma occurring in other sites may also be associated with this disease.

At present, the pathogenesis of IgG4-RD developing into lymphoma is still unclear. Studies have shown that it may be related to chronic antigenic stimulation that drives lymphoid proliferation, and the residue of reactive lymphoid follicles may provide conditions for malignant development (8). Yuka (11) et al. reported a patient with diffuse large B cell lymphoma that developed from IgG4-RD and concluded that chromosome t (14; 19) (q32; Q13.1) translocation may be associated with malignant transformation. Ohno (13) et al. believe that IgG4+ marginal zone lymphoma is characterized by the up-regulation of Th2 and regulatory cytokines, and which is similar to IgG4-RD, but different from IgG4− marginal zone lymphoma, suggesting that the pathogenesis of IgG4+ lymphoma might be different from IgG4− lymphoma. Adzavon (14) et al. preliminarily detected differentially expressed genes and pathways in IgG4-related dacryoadenitis and lymphoma using gene expression profile microarray analysis, which mainly focused on the B cell receptor signaling pathway, the NF-κB signaling pathway, FcγR mediated phagocytosis, the FcεRI signaling pathway, the EB virus infection signaling pathway, and the cancer signaling pathway.

Due to insufficient understanding of IgG4+ lymphoma and the low incidence of IgG4-RD developing into lymphoma, a large number IgG4+ lymphoma cases could not be compared with IgG4− lymphoma. The results of this study showed that there were significant differences between the two groups in laterality, preoperative glucocorticoid history, nerve thickening, serum IgG4 and prognosis. These results indicated that IgG4− lymphoma was most common in monocular disease, while IgG4+ lymphoma was more common in binocular disease, which is consistent with reported literature (7). Preoperative glucocorticoid administration was more common in IgG4+ lymphoma, which may be related to the previous history of IgG4-related ocular disease. And the expression of serum IgG4 was higher in IgG4+ lymphoma than IgG4− lymphoma. IgG4+ lymphomas showed more nerve thickening and extraocular muscle thickening on imaging, which may be a specific way to differentiate the two groups of disease. However, only ocular nerve thickening was statistically significant between the two groups, and there was no statistically significant difference in extraocular muscle thickening. Due to the small sample size and the subjectivity of imaging evaluation, this result needs to be further verified. The number of deaths was more in the IgG4− group, which may be due to a higher degree of malignancy in the cases of diffuse large B-cell lymphoma, NK/T cell lymphoma, and mantle cell lymphoma. Among these, four cases died of recurrent lymphoma, which led to a worse prognosis in the IgG4− group. However, the above results may indicate differences in pathogenesis and disease characteristics between IgG4-positive and IgG4-negative lymphoma.

Kubota (15) et al. analyzed 114 cases of ocular adrenal marginal B-cell lymphoma, and found that IgG4+ cases accounted for 9%, and the serum IgG, IgG1, IgG4, IgE, and soluble interleukin-2 receptors in the IgG4+ group were significantly higher than in the IgG4− group. These results suggest that IgG4+ lymphoma may have similar histological and serological characteristics as IgG4 related ocular disease. In our study, laboratory indicators including C3, C4, RF, ASO, CRP, IgA, IgM, IgG, IgG1, IgG2, and IgG3 were not significantly different between the two groups, which needed to be further verified.

Lee (16) et al. analyzed three cases of IgG4+ lymphoma and 40 cases of IgG4− lymphoma, and the results showed that the survival period for IgG4− lymphoma was longer, but this result was not statistically significant. And there was no statistically significant difference in event-free cumulative percentages between the IgG4+ and IgG4− groups in our study, which indicated that IgG4 positive expression may not have a significant effect on the prognosis of MALT lymphoma. However, this results may be influenced by the small sample size and short follow-up time. To determine whether IgG4+ expression has an impact on the prognosis of lymphoma, further studies may need to be conducted with a longer follow-up period.

In conclusion, the clinical features and prognosis of IgG4 positive and IgG4 negative lacrimal lymphoma were analyzed contrastively. The results of this study showed that binocular disease, ocular nerve thickening and the higher expression of serum IgG4 were more common in IgG4-positive lacrimal lymphoma than IgG4-negative lacrimal lymphoma. The pathogenesis and disease characteristics of IgG4-positive lacrimal lymphoma may differ from IgG4-negative lacrimal lymphoma, but the positive expression of IgG4 may not have significant influence on the recurrence of lacrimal lymphoma.

## Data Availability Statement

The original contributions generated for this study are included in the article/supplementary material, further inquiries can be directed to the corresponding author/s.

## Ethics Statement

The studies involving human participants were reviewed and approved by Ethics Committee of Beijing Tongren Hospital. The patients/participants provided their written informed consent to participate in this study.

## Consent for Publication

All authors read and approved the final manuscript to public.

## Author Contributions

RL analyzed and wrote the manuscript. NW, JW, and JL helped collect data. JM, XG, and JZ read and criticized the manuscript. All authors critically read and edited the manuscript and read and approved the final manuscript.

## Conflict of Interest

The authors declare that the research was conducted in the absence of any commercial or financial relationships that could be construed as a potential conflict of interest.

## Abbreviations

Ig: immunoglobulin
MALT: mucosa-associated lymphoid tissue
HPF: high power field
MRI: magnetic resonance imaging
CT: computed tomography
RF: rheumatoid factor
CRP: c-reactive protein
ASO: hemolysin against streptococcus
IgG4-RD: IgG4-related disease.

## References

[1] Ahmed S Shahid RK Sison CP Fuchs A Mehrotra B. Orbital lymphomas: a clinicopathologic study of a rare disease. Am J Med Sci. (2006) 331:79–83. 10.1097/00000441-200602000-00013 16479179

[2] Svendsen FH Heegaard S. Lymphoma of the eyelid. Surv Ophthalmol. (2017) 62:312–31. 10.1016/j.survophthal.2016.11.009 27894880

[3] Jung H Yoo HY Lee SH Shin S Kim SC Lee S. The mutational landscape of ocular marginal zone lymphoma identifies frequent alterations in TNFAIP3 followed by mutations in TBL1XR1 and CREBBP. Oncotarget. (2017) 8:17038–49. 10.18632/oncotarget.14928 28152507 PMC5370020

[4] Kalogeropoulos D Papoudou-Bai IA Kanavaros P Kalogeropoulos C. Ocular adnexal marginal zone lymphoma of mucosa-associated lymphoid tissue. Clin Exp Med. (2018) 18:151–63. 10.1007/s10238-017-0474-1 28939925

[5] Andrew NH Sladden N Kearney DJ Selva D. An analysis of IgG4-related disease (IgG4-RD) among idiopathic orbital inflammations and benign lymphoid hyperplasias using two consensus-based diagnostic criteria for IgG4-RD. Br J Ophthalmol. (2015) 99:376–81. 10.1136/bjophthalmol-2014-305545 25185258

[6] Andrew N Kearney D Selva D. IgG4-related orbital disease: a meta-analysis and review. Acta Ophthalmol (Copenh). (2013) 91:694–700. 10.1111/j.1755-3768.2012.02526.x 22963447

[7] Oleś K Składzień J Szczepański W Okoń K Leszczyńska J Bojanowska E. Immunoglobulin G4-related disease (IgG4-RD) in the orbit: mucosa-associated lymphoid tissue (MALT)-type lymphomas. Med Sci Monit. (2015) 21:1043–50. 10.12659/MSM.893043 25858500 PMC4403377

[8] Sohn EJ Ahn HB Roh MS Jung WJ Ryu WY Kwon YH. Immunoglobulin G4 (IgG4)-positive ocular adnexal mucosa-associated lymphoid tissue lymphoma and idiopathic orbital inflammation. Ophthalmic Plast Reconstr Surg. (2018) 34:313–9. 10.1097/IOP.0000000000000965 28749851

[9] Go H Kim JE Kim YA Chung HK Khwarg SI Kim CW. Ocular adnexal IgG4-related disease: Comparative analysis with mucosa-associated lymphoid tissue lymphoma and other chronic inflammatory conditions. Histopathology. (2012) 60:296–312. 10.1111/j.1365-2559.2011.04089.x 22211288

[10] Cheuk W Yuen HK Chan AC Shih LY Kuo TT Ma MW. Ocular adnexal lymphoma associated with IgG4+ chronic sclerosing dacryoadenitis: a previously undescribed complication of IgG4-related sclerosing disease. Am J Surg Pathol. (2008) 32:1159–67. 10.1097/PAS.0b013e31816148ad 18580683

[11] Kawaji Y Nagata H Muramatsu A Kuriyama K Ohshiro M Hirakawa Y. Diffuse large B cell lymphoma with chromosomal translocation t(14;19)(q32;q13) occurring in IgG4-related disease. Ann Hematol. (2019) 98:1785–7. 10.1007/s00277-019-03688-w 31111176

[12] Nishida K Sogabe Y Makihara A Senoo A Morimoto H Takeuchi M. Ocular adnexal marginal zone lymphoma arising in a patient with IgG4-related ophthalmic disease. Mod Rheumatol. (2019) 29:383–7. 10.1080/14397595.2016.1216733 27686866

[13] Ohno K Sato Y Ohshima K Takata K Miyata-Takata T Takeuchi M. A subset of ocular adnexal marginal zone lymphomas may arise in association with IgG4-related disease. Sci Rep. (2015) 5:13539. 10.1038/srep13539 26311608 PMC4550912

[14] Adzavon YM Zhao P Zhang X Liu M Lv B Yang L. Genes and pathways associated with the occurrence of malignancy in benign lymphoepithelial lesions. Mol Med Rep. (2018) 17:2177–86. 10.3892/mmr.2017.8149 29207199 PMC5783467

[15] Kubota T Moritani S Yoshino T Nagai H Terasaki H. Ocular adnexal marginal zone B cell lymphoma infiltrated by IgG4-positive plasma cells. J Clin Pathol. (2010) 63:1059–65. 10.1136/jcp.2010.082156 20980530 PMC2991078

[16] Lee MJ Kim N Choe JY Khwarg SI Jeon YK Choung HK. Clinicopathological analysis of ocular adnexal extranodal marginal zone B-cell lymphoma with IgG4-positive cells. PLoS ONE. (2015) 10:e0131458. 10.1371/journal.pone.0131458 26111022 PMC4481416

